# Reticular Skin Rash as an Adverse Effect of 5-Azacitidine

**DOI:** 10.7759/cureus.24228

**Published:** 2022-04-18

**Authors:** Parul Verma, Usha Chandra, Prakriti Shukla, Shailendra P Verma, Swastika Suvirya

**Affiliations:** 1 Department of Dermatology, Venereology and Leprosy, King George's Medical University, Lucknow, IND; 2 Department of Dermatology, Venereology and Leprosy, Lala Lajpat Rai Medical College, Meerut, IND; 3 Department of Dermatology, Venereology and Leprosy, Hind Institute of Medical Sciences, Sitapur, IND; 4 Department of Clinical Hematology, King George's Medical University, Lucknow, IND

**Keywords:** acute myeloid leukemia, adverse cutaneous drug reaction, reticular rash, generalised skin rash, azacitidine

## Abstract

Azacitidine is a hypomethylating agent used for the treatment of patients with myelodysplastic syndrome (MDS). It has been approved by the Food and Drug Administration (FDA) and the European Medicines Agency for the treatment of MDS and is also indicated for the treatment of acute myeloid leukemia (AML). Injection site erythema, ecchymosis, and petechiae are some of the common cutaneous adverse reactions associated with azacitidine. This article describes a rare adverse cutaneous drug reaction with azacitidine in the form of a reticular generalized skin rash in a 28-year-old female with AML.

## Introduction

Myelodysplastic syndromes (MDS) refer to a heterogeneous group of hematopoietic disorders characterized by one or more peripheral blood cytopenias due to a decrease in bone marrow function. MDS can be indolent or rapidly progressive, with the risk of evolution into acute myeloid leukemia (AML) [1]. Azacitidine is one of the approved hypomethylating agents for the management of MDS [2]. We describe a young female patient who was treated with intravenous azacitidine and developed a drug-induced reticular skin rash, which is an exceedingly rare side effect of azacitidine.

## Case presentation

A 28-year-old female presented with a two-month history of recurrent low-grade fever, decreased appetite, and generalized weakness. There was no past history of known allergic reactions or hypersensitivity to any drug. The patient had splenomegaly 3 cm below the left costal margin, along with anemia, thrombocytopenia, and leucocytosis. Her bone marrow aspiration showed the presence of 70% blasts with suppression of all hematopoietic lineages. Flow cytometry from peripheral blood confirmed the diagnosis of AML. Karyotype was normal, and PCR was negative for AML-eight-twenty one oncoprotein (ETO), promyelocytic leukemia-retinoic acid receptor alpha (PML-RARA), FMS-like tyrosine kinase 3 (FLT-3), nucleophosmin (NPM1), and receptor tyrosine kinase (c-KIT) mutations. She was diagnosed as a case of acute myeloid leukemia (AML) under the hemato-oncology division of our hospital.

The patient was planned for intravenous azacitidine 200 mg/day on the first day (day 1), followed by 100 mg/day for six days (day 2 to day 7). Azacitidine was considered for induction therapy as the patient had a fever, active pulmonary infection, and poor performance status. Initially, 3+7 induction was not possible, so it was planned after control of infection and improvement of performance status. Also, in view of underlying severe thrombocytopenia and similar efficacy, the intravenous route of administration of azacitidine was preferred.

**Figure 1:**
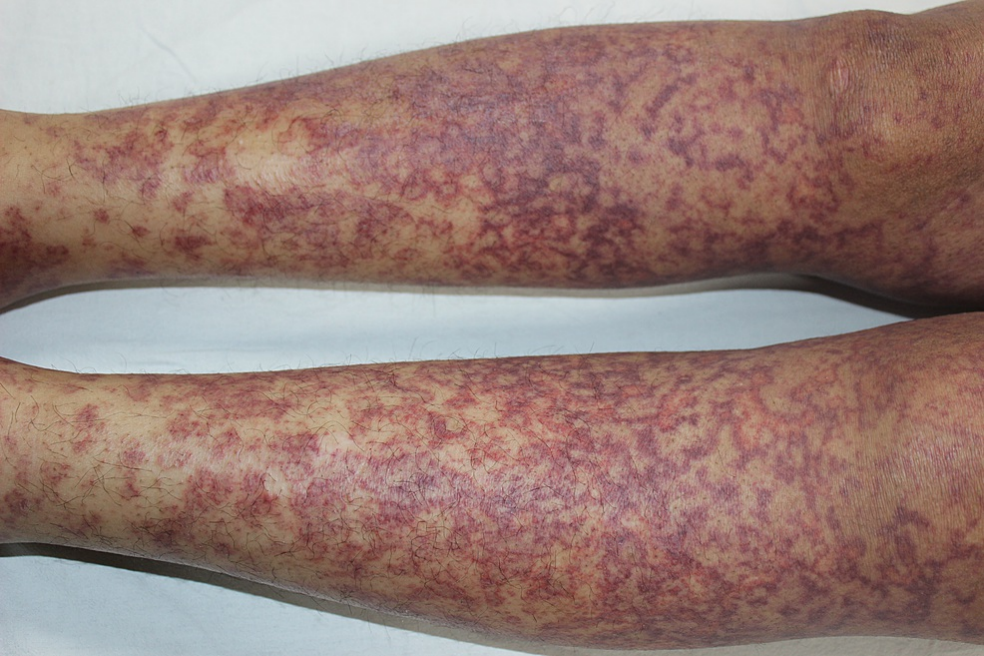
Multiple erythematous-to-violaceous reticular maculopapular lesions over bilateral legs.

After two days of starting azacitidine, the patient developed an itchy erythematous-to-violaceous reticular maculopapular rash of varying sizes, ranging from 2 mm to 30 mm in diameter (Figure 1).

**Figure 2:**
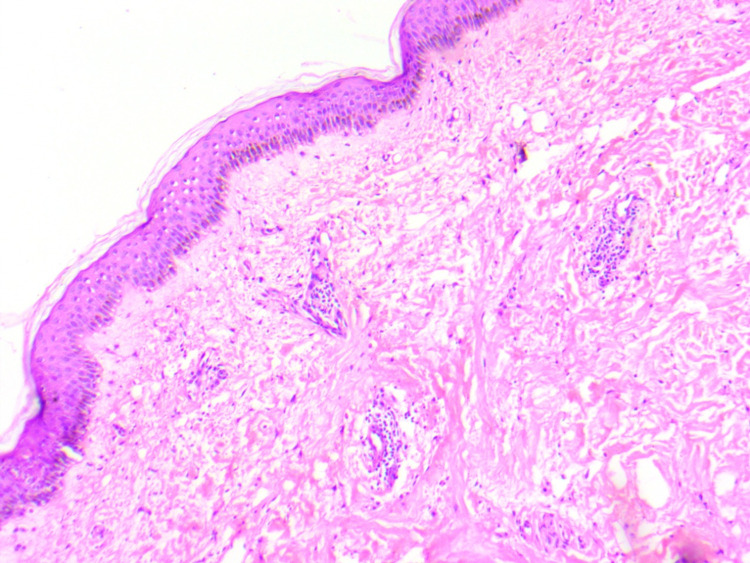
Biopsy section shows keratinized stratified squamous epithelium with an underlying sub-epithelial zone of necrosis and collagenolysis (hematoxylin & eosin, 100×).

The lesions started over the bilateral upper and lower limbs and gradually involved the trunk and back. These were non-blanchable, and there was no mucosal involvement. No injection-site reaction was observed. Systemic examination was also not contributory. Skin biopsy from erythematous plaque showed an area of collagen necrosis along with a mild perivascular chronic inflammatory infiltrate and no eosinophils or vasculitis (Figure 2).

The Naranjo adverse drug reaction probability scale revealed a total score of 5 (the adverse event occurred after the suspected drug was administered-score = 2, the adverse reaction improved when the drug was discontinued-score = 1, and the adverse event was confirmed by objective evidence-score = 1), which suggested that there is a probability of the adverse event being related to the prescribed drug. Based on the clinical and histopathological findings, a diagnosis of an azacitidine-induced reticular maculopapular rash was made. Azacitidine was stopped and injections of decitabine were started along with other supportive treatments. Bland emollient with antihistamine was prescribed for her cutaneous symptoms. The patient improved remarkably in two weeks and the lesions subsided with minimal post-inflammatory hyperpigmentation.

## Discussion

5-Azacitidine, a nucleoside metabolic inhibitor, is an Food and Drug Administration (FDA)-approved drug for the treatment of adult patients with acute myeloid leukemia. The adverse reactions to azacitidine include nausea, vomiting, cytopenia, pyrexia, diarrhea, fatigue, constipation, pain in extremities, and arthralgia. Usually, these adverse events occur transiently in the early treatment cycle and resolve during ongoing therapy. Common cutaneous adverse reactions by intravenous or subcutaneous route include injection site erythema [3] (46%-72%), ecchymosis, and petechiae, which may be due to inflammatory responses to drugs that rarely require corticosteroids and/or antihistamines. Few patients have been shown to develop a rash, nodule, Nicolau syndrome, Sweet syndrome [4], and Pyoderma gangrenosum [5] at the injection site. These may be annoying to the patient as they are associated with pain, itching, burning, and cosmetic concern.

Other than localized reactions at the injection site, few generalized reactions are reported in the literature. Keating et al. reported a severe phototoxic reaction due to subcutaneous 5-azacitidine [6]. A case series of azacitidine-associated generalized urticarial skin rash has been reported in the literature, which resolved effectively after the use of concomitant low-dose oral steroids [7]. Lunge et al. also described a biopsy-proven case of leucocytoclastic vasculitis due to azacitidine, which resolved after discontinuation of the drug [8]. Toxic erythema of chemotherapy and maculopapular erythematous rash have also been found to be associated with this drug [9,10].

To the best of our knowledge, this is the first interesting and worth-sharing case report of a reticular appearance of a rash associated with azacitidine. The Naranjo scale was used in this case to determine the likelihood (unlikely, possibly, probably, or definitely) if the drug was associated with the adverse drug reaction, and it was found to have a probable likelihood [11].

## Conclusions

Azacitidine is an FDA-approved drug for the treatment of adult patients with acute myeloid leukemia. Other than the common systemic adverse effects associated with azacitidine, such as nausea, vomiting, cytopenia, and arthralgia, which are transient, injection site erythema, ecchymosis, and petechiae constitute some frequently encountered cutaneous adverse effects. The present case represents a rare adverse cutaneous drug reaction with azacitidine in the form of a reticular generalized skin rash in a young female with AML.
